# Differential activity of novel gametocytocidal compounds: drug mode-of-action and *ex vivo* efficacy

**DOI:** 10.1186/1475-2875-13-S1-P13

**Published:** 2014-09-22

**Authors:** Lyn-Marie Birkholtz, Didier LeRoy, Janette Reader, Mariette Botha, Dalu Mancama, Theresa L Coetzer

**Affiliations:** 1 University of Pretoria, Pretoria, South Africa; 2 Medicines for Malaria Venture, Geneva, Switzerland; 3 Council for Scientific and Industrial Research, Pretoria, South Africa; 4 University of Witwatersrand, Johannesburg, South Africa

## 

As per the 2007 Global Malaria Eradication Plan, malaria transmission blocking is seen as key to malaria elimination strategies. A highlight that has emerged from gametocytocidal assays to identify novel compounds with malaria transmission blocking ability is that, unlike asexual-based assays, greater variability in end-point readout may exist between these assays that interrogate different parasite biological functions. Drug mode-of-action is likely to be an important factor on this outcome. Such variability may be mitigated by screening compounds based on similar pharmacophores in series. One of the major concerns with the current assay platforms is their inability to be robustly used to screen variant pharmacophores accurately as the different assay principles may interrogate different biological functions. As such, compounds targeting a specific biological pathway may in extreme cases either fail in a certain assay, or by contrast, may be flagged as false positives. Taking assay platform differences into account, and relying on good intra-assay variability for each assay optimized in our laboratories, the ATP, pLDH, luciferase reporter and PrestoBlue™ assays were compared in context of a blinded MMV 10-compound set. All the assays were performed in parallel on the same gametocyte population (except for the luciferase reporter lines). The remaining parameters for each assay were all comparable. In each case, the assay was performed for 48 h of continuous drug pressure for at least three replicates. Although direct comparison of absolute inhibition values are difficult between assay platforms, similar trends were observed including comparative performance of the luciferase marker assay and the PrestoBlue™ assay for e.g. DHA and Methylene blue. Interestingly, the ATP assay could not detect any inhibitory activity for some quinoline family members and may therefore be more sensitive in indicating the inability of these compounds to inhibit gametocytes. Data from the luciferase reporter assays for these compounds indicate that the compounds are indeed more active against early stage gametocytes. The signal obtained for these compounds in the PrestoBlue™ and pLDH assays may therefore rather reflect the inability of these assays in discriminating activity of compounds against earlier stages of gametocytes, whereas the ATP assay more accurately reflect these compounds’ activity. Whilst this data is informative from a biological perspective and may provide indications of the drug mode of action, it does highlight the care that has to be taken in screening platforms where compounds may be falsely assigned activity (or lack thereof) based on a single assay.

